# New Targets for Schizophrenia Treatment beyond the Dopamine Hypothesis

**DOI:** 10.3390/ijms18081689

**Published:** 2017-08-03

**Authors:** Albert C. Yang, Shih-Jen Tsai

**Affiliations:** 1 Department of Psychiatry, Taipei Veterans General Hospital, Taipei 112, Taiwan; accyang@gmail.com; 2 Division of Psychiatry, School of Medicine, National Yang-Ming University, Taipei 112, Taiwan; 3 Division of Interdisciplinary Medicine and Biotechnology, Beth Israel Deaconess Medical Center/Harvard Medical School, Boston, MA 02215, USA

**Keywords:** schizophrenia, dopamine hypothesis, novel treatment target

## Abstract

Schizophrenia has been primarily associated with dopamine dysfunction, and treatments have been developed that target the dopamine pathway in the central nervous system. However, accumulating evidence has shown that the core pathophysiology of schizophrenia might involve dysfunction in dopaminergic, glutamatergic, serotonergic, and gamma-aminobutyric acid (GABA) signaling, which may lead to aberrant functioning of interneurons that manifest as cognitive, behavioral, and social dysfunction through altered functioning of a broad range of macro- and microcircuits. The interactions between neurotransmitters can be modeled as nodes and edges by using graph theory, and oxidative balance, immune, and glutamatergic systems may represent multiple nodes interlocking at a central hub; imbalance within any of these nodes might affect the entire system. Therefore, this review attempts to address novel treatment targets beyond the dopamine hypothesis, including glutamate, serotonin, acetylcholine, GABA, and inflammatory cytokines. Furthermore, we outline that these treatment targets can be possibly integrated with novel treatment strategies aimed at different symptoms or phases of the illness. We anticipate that reversing anomalous activity in these novel treatment targets or combinations between these strategies might be beneficial in the treatment of schizophrenia.

## 1. Introduction

Since the discovery of chlorpromazine to treat schizophrenia, studies have been focusing on dopamine dysfunction, particularly in the mesolimbic dopamine pathway, which increases dopamine synthesis and release capacity, and can lead to psychosis [1,2].

Many studies have indicated that schizophrenia is a disorder of the dopamine signal system. Dopamine was initially reported to be related to motor function, but was then found to be associated with reward and motivation in animal studies [3]. Central nervous system stimulants, such as amphetamine, can increase dopamine release and may cause psychotic symptoms. The potency of an antipsychotic is proportional to its ability to antagonize dopamine D2/3 receptors [4]. In early imaging studies based on positron emission tomography (PET), patients with schizophrenia showed increased dopamine activity in the striatum [5] and the midbrain origin of neurons [6] compared with the controls. In addition, this increase was observed in patients having a high risk of schizophreniform psychosis [7] and was specifically linked to those who later developed psychosis [8]. Therefore, dopaminergic dysfunction is proposed as a final common pathway leading to psychosis in schizophrenia [4]. However, the mechanism through which increased dopamine synthesis and release capacity leads to the surfacing of the symptoms and signs of psychosis remains unclear [2,9].

The dopamine hypothesis, which states that the dysregulation of the dopaminergic system is etiologic for schizophrenia, is among the most enduring biological theories in psychiatry. Although variations within genes related to dopaminergic functioning have been associated with schizophrenia, an aggregate test of variations—by using the largest publicly available schizophrenia dataset—has not been conducted. Dopamine dysfunction and its treatment are not sufficient to explain the psychopathology of schizophrenia and its treatment outcomes. In addition to the devastating symptoms of psychosis, many patients with schizophrenia have cognitive impairment. These cognitive symptoms cause substantial dysfunction and can affect their ability to work, to adhere to treatment, and to apply social skills. In addition to the dopamine hypothesis, novel targets have been proposed for schizophrenia treatment. This review intends to address the dopamine hypothesis and the novel targets that have been proposed in recent years (Table 1).

## 2. Dopamine Hypothesis and Beyond

The dopamine hypothesis of schizophrenia was first proposed in the 1960s when the first antipsychotic chlorpromazine was found to successfully treat the positive symptoms of patients with schizophrenia. Since then, the development of newer antipsychotics has generally been following the dopamine hypothesis that patients with schizophrenia have increased dopaminergic activity, which can be normalized using dopamine antagonists, particularly the dopamine D2 receptor antagonist.

The dopamine D2 receptor is a G protein-coupled receptor, which is a common target for antipsychotic drugs. The antagonism of dopamine D2 receptor in the mesolimbic pathway is thought to be the main mode of action of antipsychotic medication in treating psychotic symptoms. However, a dopamine receptor antagonist is not clinically effective at treating cortical-related symptoms, such as cognitive deficits, in schizophrenia. Although the exact mechanism underlying these cognitive deficits remains largely unknown, factors such as deficits in cortical dopamine function [10], dysfunction in the NMDA receptor [11], or synaptic elimination [12], are likely to play a contributing role.

Molecular imaging studies have supported an association of increased subcortical dopamine transmission with the positive symptoms of schizophrenia, with the limitation that this finding is not pathognomonic, due to the neurochemical heterogeneity of the populations of patients with schizophrenia. Although hyperactivity in subcortical dopaminergic function contributes substantially to aberrant salience (possibly manifesting in positive symptoms), the original dopamine hypothesis must be extended to include contributions of other neurotransmitter systems in the pathophysiology of schizophrenia [13].

### 2.1. Glutamate

Glutamate is one of the excitatory neurotransmitters and the most abundant neurotransmitter in the brain. Glutamate is mediated by *N*-methyl-d-aspartate receptors (NMDARs) [14,15], and its pathways link to the cortex, the limbic system, and the thalamus regions, which have been implicated in schizophrenia [16]. The association of glutamate dysfunction with schizophrenia could originate from the observation that the cerebrospinal fluid of patients with schizophrenia had decreased glutamate levels [17]. Furthermore, patients who abuse NMDAR antagonists, such as phencyclidine or ketamine, frequently show psychotic symptoms [18]. NMDARs bind to glutamate and its coagonists glycine or d-serine, both of which offer additional therapeutic targets. Dysfunction of glutamatergic neurotransmission can be a promising treatment target of schizophrenia for its essential role in the pathophysiology of schizophrenia in terms of negative symptoms and cognitive impairment.

### 2.2. Serotonin

The serotonin (5-hydroxytryptamine; 5-HT) hypothesis of schizophrenia is based on the early studies of interactions between the hallucinogenic drug lysergic acid diethylamide (LSD) and 5-HT [19]. Observations of the psychotogenic effects of LSD and the antipsychotic effects of serotonin-dopamine antagonists, such as clozapine and risperidone, have led to increased interest in the interaction between these two neurotransmitter systems as a possible pathophysiological target in schizophrenia [20].

Serotonin antagonists ameliorate the extrapyramidal effects of antipsychotics. However, direct evidence of serotonergic dysfunction in the pathogenesis of schizophrenia is not yet available; specific serotonin receptors continue to be a focus of interest (particularly 5HT-3 and 5HT-6) in schizophrenia [21].

### 2.3. Acetylcholine

A significant proportion of patients with schizophrenia are heavy smokers. The high rate and heavy level of smoking observed in this population may be related to the illness or its treatment [22]. Studies have suggested the effect of altered brain muscarinic activity in schizophrenic patients [23,24]. Patients reported that they smoked for sedation to reduce negative symptoms and to counteract medication side effects [25,26]. These observations may reflect the patients’ efforts to overcome the deficit in nicotinic cholinergic receptors.

Patients with schizophrenia were found to have a poor inhibition of P50-evoked responses to repeated auditory stimuli, which may reflect impaired sensory gating. However, the effect of smoking on the reversal of diminished auditory sensory gating in schizophrenia may be attenuated due to the desensitization of the nicotine receptor. This observation has been associated with the chromosome 15q14 locus of the α-7 nicotinic receptor gene [27] and has motivated studies on promuscarinic agents, such as α-7 nicotinic agonists, in treating certain symptoms of schizophrenia [28]. Thus, the nicotinic cholinergic-mediated inhibitory process may represent a potential treatment target in schizophrenia.

### 2.4. Gamma-Aminobutyric Acid

GABA is the major inhibitory neurotransmitter in the central nervous system. A model has suggested the role of GABA (including GABA–dopamine interactions) in schizophrenia [29,30]. Alterations in the GABA neurotransmitter system have been reported in clinical and basic neuroscience schizophrenia studies as well as in animal models, and may be involved in the pathophysiology of schizophrenia. The chandelier subtype of parvalbumin-positive GABA neurons may be particularly affected by, and specific to schizophrenia [31].

GABA interneurons are central to brain's rhythm-generating networks, and synchrony of neural oscillations is a fundamental mechanism for the memory, perception and consciousness [32]. GABA abnormalities may underlie alterations in neural synchrony [32], abnormal gamma oscillations [33], and working memory impairments, which are observed in schizophrenia [34]. In clinical studies, the adjunctive GABA agonists has been shown to be effective in improving core schizophrenia symptoms [35]. However, it is unclear that how GABA interacts with other neurotransmitter systems, and requires further studies to elucidate the potential therapeutic role of GABA in schizophrenia treatment.

### 2.5. Inflammation

Inflammation and oxidative stress are other areas of interest in studies on the pathophysiology of schizophrenia [36,37]. Alterations in the complement system, which mediates innate immunity, have been implicated in schizophrenia, with observations of increases in C1, C3 and C4 complement protein activity [38,39]. Complement proteins may “tag” synapses for phagocytosis by activated microglia, leading to accelerated pruning of synapses [40,41].

An example of an inflammatory model of psychotic disorders is the anti-NMDAR encephalitis syndrome [42]. In this syndrome, schizophrenia-like features, including catatonic symptoms and autonomic dysfunction, may be combined with an increase in the level of NMDAR autoantibodies; immunotherapy is helpful in its treatment [43]. Another treatable immune model of schizophrenia is gluten sensitivity, which may involve an increase in the level of transglutaminase antibodies; patients with gluten sensitivity may benefit from gluten-free diets [44].

### 2.6. Summary of the Hypothesis beyond Dopamine Mechanism

In summary, the core pathophysiology of schizophrenia might primarily involve anomalies of the dopaminergic system, and accumulative evidence suggests that glutamatergic, serotonergic, and GABA alterations can lead to aberrant functioning of interneurons, which is manifested as cognitive, behavioral, and social dysfunction through altered functioning of a broad range of macro- and microcircuits. Genetic and early-life environmental risk factors and their interactions can contribute to these abnormalities. Unfortunately, none of the single neurotransmitter systems could explain the full picture of the heterogeneity of schizophrenia symptoms. Intriguingly, the dopaminergic system may interact with other neurotransmitter pathways. For example, postsynaptic density has been suggested to be involved in schizophrenia, and is related to dopaminergic and glutamatergic systems [45]. The interactions between neurotransmitter systems lead to complex and diverse patterns of mechanisms potentially involved in the pathophysiology of schizophrenia [46]. Thus, the treatment strategy that exclusively targets a single neurotransmitter system is less likely to be successful.

Therefore, identifying the role of novel treatment agents in such complex neurotransmitter networks could be important for understanding their mechanisms of action. Such interactions can be possibly modeled as nodes and edges using graph theory, a novel tool in computational biology for studying complex network interactions. In this approach, dopaminergic, glutamatergic or serotonergic systems may represent multiple nodes connected at a central hub; imbalance within any of these nodes might affect the entire system [47]. Reversal of anomalous activity in any of these nodes, or any combinations thereof, might have beneficial effects on the entire system.

## 3. Novel Treatment Strategies

The relationship between neurobiological findings and clinical symptoms of schizophrenia could lead to the development of novel drug treatments targeting different phases of the illness [48,49,50]. In addition, improvement in the understanding of the pathophysiology of schizophrenia enables us to define potential therapeutic targets more systematically [36,37,51,52] and utilize more actionable biomarkers [53]. The development of antipsychotics was supposed to be disease-specific and it targeted the core pathology of schizophrenia encompassing both positive and negative symptom domains. However, current antipsychotics usually show higher efficacy in treating positive symptoms, such as hallucinations or delusions, than negative symptoms or cognitive deficits [54,55]. Therefore, medication that can specifically target negative or other nonpsychotic symptoms may have substantial clinical applications, either being used as an add-on adjuvant or a stand-alone treatment. Additionally, recent discoveries in the genetic susceptibility of schizophrenia may implicate in the treatment of the illness. In the context of the complex heterogeneity of schizophrenia, we discuss these potential therapeutic approaches in this review.

### 3.1. Specific Treatments for Negative Symptoms

A major factor for severe disability in patients with schizophrenia is its persistent and deteriorating negative symptoms. Nevertheless, few current treatments have shown efficacy in this domain [56]. In recent years, several novel strategies have been evaluated [57], including glutamatergic receptors with glycine transporter inhibitors (e.g., bitopertin) [58] and metabotropic M2/M3 agonists (e.g., pomeglumetad methionil) [59]. These studies have yielded positive results, which have not yet been confirmed in subsequent studies. In addition to agents targeting the glutamatergic system, treatments targeting nicotinic and muscarinic cholinergic agents [24,60], antibiotics (e.g., minocycline) [61], and hormones (e.g., oxytocin [62], estrogen receptor modulator raloxifene [63], and pregnenolone [64]) have also emerged in recent years to treat negative symptoms of schizophrenia. However, additional studies are warranted to evaluate their efficacy and consistency of the treatment response.

### 3.2. Specific Treatments for Cognitive Deficits

Cognitive impairment associated with schizophrenia is a significant predictor of social and vocational disability [65]. Currently, the exact mechanisms underlying cognitive impairments in schizophrenia are not fully understood, and reduced brain function in the prefrontal cortex (known as hypofrontality) has often been observed in patients with schizophrenia [66]. Some pharmacological targets, such as those targeting the nicotine system, are currently being investigated [54,67,68]. However, no consistent benefits from any of these strategies have been demonstrated.

### 3.3. Specific Treatment for Different Phases of the Illness

Pathophysiological processes may vary across different stages of schizophrenia, but current pharmacological treatment does not typically differ during the course of the illness [69]. Several risk factors contribute to the clinical manifestations of the illness. Approaches toward prevention are focused on the early identification of those at risk and the development of safe and effective interventions that can eliminate specific risks [70,71]. Treatments that can potentially prevent deteriorative brain processes and approaches to reverse them are currently being evaluated [72].

## 4. Novel Treatment Targets

### 4.1. Dopaminergic Antagonists and Stabilizers

All current antipsychotic therapies have been developed based on the antagonism of dopaminergic receptors or dopamine “stabilizers” including D2/D3 partial agonists, such as the currently available aripiprazole. An exception is the recent introduction of pimavanserin as an antipsychotic in Parkinson’s disease [73]. The development of antipsychotics targeting other neurotransmitters may raise the hope of addressing the frequent problem of nonadherence, which is a significant roadblock in the recovery of many patients with schizophrenia.

### 4.2. Glutamatergic Agents

Increasing attention has been focused on glutamatergic dysfunction as a pathophysiological mechanism in schizophrenia, and various therapeutic approaches for schizophrenia are being evaluated. Among potential targets related to glutamatergic dysfunction, agents stimulating NMDARs have received the most attention. Because direct NMDAR agonists are neurotoxic, efforts to stimulate NMDARs indirectly by using glycinergic agents (e.g., serine and cycloserine) and glycine transport (GlyT1) inhibitors (e.g., bitopertin) have been extensively studied. Glycine and d-serine bind to the NMDAR site as coagonists; thus, an increase in glycine availability by GlyT1 inhibitors is shown to be effective in preclinical studies [74]. However, only moderate benefits on negative symptoms of schizophrenia were demonstrated in the clinical trial [58].

Metabotropic receptors 1 receptor antagonists have been found to be potential targets for positive symptoms of schizophrenia in animal models [75,76], but the development of related treatment agents have not yet reached clinical trials. Additionally, metabotropic 2 and 3 receptor agonists (e.g., pomaglumetad methionil) that reduce excessive glutamate release have also received considerable attention [77]. Similar to the development of bitopertin, initial studies with pomaglumetad methionil were positive [78], whereas other studies have not confirmed its efficacy [59,79]. These agents might be effective only in the earlier stages of schizophrenia [80], or in individuals having specific genetic polymorphisms, such as in neuregulin [81]. These preliminary findings are promising, but require further evaluation through clinical trials.

### 4.3. Serotonin Agents

Second-generation antipsychotics having serotonin–dopamine antagonism provides certain protection against extrapyramidal symptoms, although these agents, except for clozapine, do not provide additional efficacy relative to the first-generation antipsychotics [54]. Alternative 5-HT approaches in schizophrenia under investigation include the use of 5-HT1A agonists, 5-HT reuptake inhibitors, 5-HT2C antagonists and agonists, 5-HT3 antagonists, 5-HT6 antagonists, and 5HT7 antagonists either individually or in combination with D2 antagonism or 5-HT2A antagonism or both [82]. Although agents with D2/5-HT2A antagonism and 5-HT1A partial agonism are currently available, efforts to develop agents with more potent 5-HT1A activity (targeting negative and cognitive symptoms) with better tolerability are ongoing. Recently, there is a renewed interest in the development of 5-HT3 antagonists (ondansetron, tropisetron and granisetron) as adjunctive agents for negative and cognitive symptoms [83,84,85,86]. For example, Ondansetron is a 5-HT3 receptor antagonist widely used to prevent nausea and vomiting in patients receiving chemotherapy for cancer and is also potentially related to anti-inflammatory treatment strategy for schizophrenia. Adjunctive ondansetron has now entered a phase III trial for improving negative symptoms in schizophrenia [82,86]. Lurasidone, the most recently introduced antipsychotic, has potent 5-HT7 antagonism and may have clinical implications for the mood components of psychosis.

### 4.4. Gamma-Aminobutyric Acid (GABA) Allosteric Modulators

GABA is implicated in the pathophysiological mechanisms of schizophrenia. Benzodiazepines (that work on GABA-A receptor allosteric sites) have often been used with antipsychotic medications in patients with schizophrenia. Selective GABA-A agonists and GABA-B antagonists, and more recently, allosteric modulators at GABA-A receptor subtypes are being evaluated in schizophrenia treatment [87].

### 4.5. Cholinergic Agonists

The cholinergic system has long been a focus of studies on the pathophysiology of schizophrenia, given the clinical observation that nicotine smoking, which most patients with schizophrenia are exposed to, has procognitive effects. Both muscarinic and nicotinic receptors have been targeted in the therapy of schizophrenia [24,88,89]. Although several agents targeting these receptors are currently in various stages of study, α-7 nicotinic and M1 muscarinic agonists and positive allosteric modulators are of greatest interest [90,91]. Nicotinic agonists principally target cognitive symptoms [68], whereas muscarinic agonists appear to address positive symptoms [92,93,94].

### 4.6. Neuropeptides

Drugs targeting the neuropeptides associated with dopaminergic and glutamatergic systems have been proposed as a potential treatment strategy for schizophrenia. Cholecystokinin agonists were among the earliest to be evaluated; however, results have been equivocal [95]. Cannabis has been implicated in schizophrenia in high doses, of which the principal psychoactive constituent is tetrahydrocannabinol (THC), and may be psychotogenic through involvement in dopaminergic, GABA, and glutamatergic neurotransmission [96,97,98]. Interestingly, another major constituent of cannabis, cannabidiol, is able to prevent psychotic-like symptoms induced by high doses of THC by acting as an indirect antagonist of cannabinoid (CB) receptors [99]. As a result, the CB receptor subfamily has received considerable attention as a potential antipsychotic target, with CB antagonists being of the highest interest.

Neurokinin-3 (NK3) receptors have broad modulatory effects on several neurotransmitter systems (including dopamine and GABA); preclinical data with NK3 receptor antagonists have suggested potential efficacy across several symptom dimensions in schizophrenia. Similarly, despite studies indicating the role of neurotensin (NT) in the pathophysiology of schizophrenia [100], the questions of which NT receptor to target and how best to do so remain controversial.

### 4.7. Anti-Inflammatory Approaches

Many epidemiological, experimental, and clinical studies have demonstrated significant associations between schizophrenia and inflammatory conditions [36]. A broad range of anti-inflammatory strategies have been evaluated in schizophrenia, but results have been inconsistent [37,101]. Anti-inflammatory approaches might be the most effective in the early period of the illness [102,103].

### 4.8. Genetic-Based Approaches

Genome-wide association studies have identified over a hundred single nucleotide polymorphisms (SNPs) associated with schizophrenia risk. Despite the fact that these SNPs account for a modest fraction of genetic predisposition and almost all of them are in the non-coding region, the identified genetic risks of schizophrenia still implicate novel treatment targets via the understanding of neurotransmitter signaling. Certain genes have been already the drug targets for schizophrenia, such as the gene coding for serine racemase (SRR), which synthesizes d-serine, and one clinical trial has shown positive results with augmenting antipsychotics [104]. Other genes, such as *DISC1*, *NOS1*, *NOS1AP*, *GRM*, *Pdxdc1*, or *ZNF804A*, have been implicated in the treatment target for the schizophrenia in preclinical studies. For example, the dopamine D2 receptor has been shown to interact with the *DISC1* protein, and disruption of this interaction with a peptide has antipsychotic-like effects in animal models [105]. More recently, the *Pdxdc1* gene is a new antipsychotic treatment target due to its modulatory effect of pre-pulse inhibition [106], a behavioral endophenotype often used to screen for antipsychotic effects [105]. Additionally, genetic risks such as copy number variants (CNV) have also been a potential treatment target of schizophrenia [107]. It is noteworthy that neurobiology involved in these genetic risks is complex and requires extensive research to elucidate their therapeutic implications.

### 4.9. Other Approaches

In addition to the aforementioned approaches, epigenetic abnormalities may be a potential target for the treatment of patients with schizophrenia [108]. Recent data have indicated that epigenetic abnormalities in the disorder are more complex and include a combination of restrictive chromatin, open chromatin, and dysfunctional communication between various epigenetic mediators, leading to faulty regulation [109,110]. Epigenetic factors can account for both genetic and environmental risks and their interactions in the pathogenesis of schizophrenia. Furthermore, epigenetic factors are modifiable, and a multitude of epigenetic pharmacological treatments are already available or in development for nonpsychiatric disorders [111]. It is noteworthy that none of epigenetic treatments have been investigated in schizophrenia, and problems such as systemic toxicity may prevent it from the clinical use [112]. Finding a suitable delivery method might be necessary to reduce side effects of epigenetic drugs in potential clinical trials [113].

## 5. Conclusions

Schizophrenia treatment based on the dopamine hypothesis has been successful. However, despite many decades of effort by both scientists and drug companies, all currently available clinical treatments still primarily target the dopamine D2 receptor. The reason behind this inconvenient result may be due to the heterogeneity of psychosis. Patients with schizophrenia exhibit marked variations in symptoms, even the biological characterization of symptom domains of schizophrenia remains unclear, and the responses to different therapeutic interventions also vary significantly. The efforts of finding homogenous groups of psychosis using biological markers such as neuroimaging or genetic data may be of help for future pharmacological studies to evaluate novel treatment strategies. Additionally, the lack of reliable animal models of psychosis also contributes to the difficulty in identifying and validating novel treatment agents of schizophrenia. Alternatively, perhaps the dopamine dysfunction is indeed the core psychopathology of schizophrenia, but because of complex interactions between dopamine and other neurotransmitter systems, the development of novel treatment targets cannot be successful without considering these network-like interactions.

The difficulty to prospectively predict individual responses to specific treatments leads to a trial-and-error therapeutic strategy. Advances in pharmacogenomics (the study of the genetic determinants of drug response) generate optimism about applying these strategies in treating schizophrenia [114,115,116]. Over a hundred compounds, which encompass a broad range of targets in schizophrenia, are currently in various stages of drug development. Although recent results with some of the most novel drug candidates have been disappointing [117], the increasing robustness of genetic findings in schizophrenia have generated optimism about developing more effective and specific treatments for this disorder [52]. Furthermore, incorporation of brain imaging markers, such as those derived from PET or functional magnetic resonance imaging, into the treatment strategy can potentially provide new opportunities for precisely treating the illness and tracking the outcome of schizophrenia [118].

## Figures and Tables

**Table 1:** Novel treatment targets for schizophrenia.

Hypothesis	Target	Strategy
Dopamine	Dopaminergic stabilizers	Improve medication adherence
Glutamate	NMDAR, AMPA receptor, or metabotropic receptors	Improve negative symptoms and cognitive impairments
Serotonin	5-HT1A agonists, 5-HT reuptake inhibitors, 5-HT2C antagonists and agonists, 5-HT3 antagonists, 5-HT6 antagonists, and 5HT7 antagonists	Reduce the extrapyramidal effects Improve negative symptoms and cognitive impairments Potential treatment for different phases of the illness
Acetylcholine	α-7 nicotinic and M1 muscarinic agonists and positive allosteric modulators	Nicotinic agonists for cognitive symptoms Muscarinic agonists for positive symptoms
Gamma-aminobutyric acid	Selective GABA-A agonists, GABA-B antagonists, and allosteric modulators at GABA-A receptor subtypes	Augmentation of psychosis treatment
Inflammation	Cytokines	Possibly the early period of the psychosis

NMDAR: *N*-methyl-d-aspartate receptors; AMPA: α-amino-3-hydroxy-5-methyl-4-isoxazolepropionic acid; 5-HT: 5-hydroxytryptamine; GABA: gamma-aminobutyric acid.
